# Supplementary material for: Boosting the electronic and catalytic properties of 2D semiconductors with supramolecular 2D hydrogen-bonded superlattices
Source: Nat Commun. 2022 Jan 26;13:510. doi: 10.1038/s41467-022-28116-y (PMC8791956; doi:10.1038/s41467-022-28116-y)
Supplement: Supplementary file 1 — Supplementary Information [file 41467_2022_28116_MOESM1_ESM.pdf]

## Supplementary Information

**Boosting the electronic and catalytic properties of 2D semiconductors with supramolecular 2D hydrogen-bonded superlattices**

Can Wang,[1] Rafael Furlan de Oliveira,[1] Kaiyue Jiang,[2] Yuda Zhao,[1] Nicholas Turetta,[1] Chun Ma,[1] Bin Han,[1] Haiming Zhang,[3] Diana Tranca,[2] Xiaodong Zhuang,[2] Lifeng Chi,[3] Artur Ciesielski[1,*] & Paolo Samorì[1,*]

[1] Université de Strasbourg, CNRS, ISIS, 8 allée Gaspard Monge, 67000 Strasbourg, France.

E-mail: ciesielski@unistra.fr, samori@unistra.fr

[2] The Meso-Entropy Matter Lab, The State Key Laboratory of Metal Matrix Composites, Shanghai Key Laboratory of Electrical Insulation and Thermal Ageing, School of Chemistry and Chemical Engineering, Frontiers Science Center for Transformative Molecules, Shanghai Jiao Tong University, Shanghai 200240, P. R. China

[3] Institute of Functional Nano & Soft Materials (FUNSOM) Jiangsu Key Laboratory for Carbon Based Functional Materials & Devices, Soochow University, Suzhou 215123, P. R. China

### Table of contents

Supplementary Figure 1. ........ S2
Supplementary Figure 2. ........ S2
Supplementary Figure 3. ........ S2
Supplementary Figure 4. ........ S3
Supplementary Figure 5. ........ S4
Supplementary Figure 6. ........ S4
Supplementary Figure 7. ........ S5
Supplementary Figure 8. ........ S6
Supplementary Figure 9. ........ S7
Supplementary Figure 10. ........ S8
Supplementary Figure 11. ........ S9
Supplementary Figure 12. ........ S10
Supplementary Figure 13. ........ S11
Supplementary Figure 14. ........ S12
Supplementary Figure 15. ........ S12
Supplementary Figure 16. ........ S13
Supplementary Figure 17........ S13
Supplementary Figure 18. ........ S14
Supplementary Table 1........ S14
Supplementary Figure 19. ........ S15
Supplementary Figure 20. ........ S16
Supplementary Figure 21. ........ S17
Supplementary Figure 22. ........ S17
Supplementary Figure 23. ........ S18
Supplementary Table 2........ S19
Supplementary Table 3........ S19

Supplementary Figure 24. ........................................................................ S19
Supplementary Figure 25. ........................................................................ S20
Supplementary References........................................................................ S21

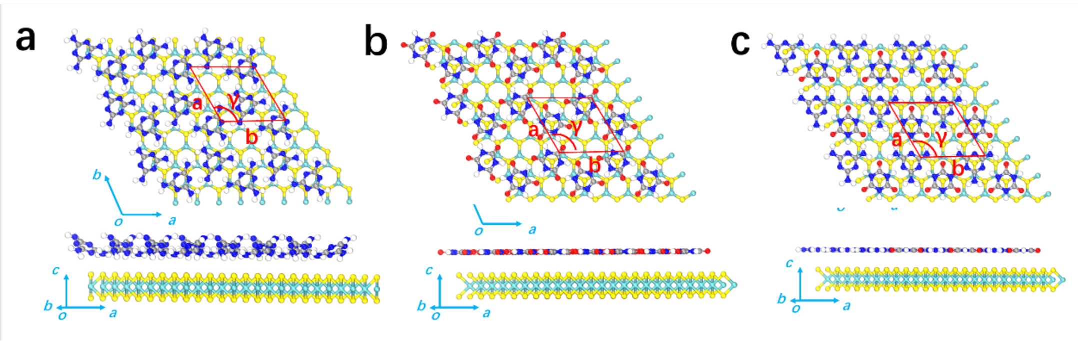


**Supplementary Figure 1: Calculated SAM structures on monolayer $MoS_2$ by DFT.** (a) M SAM on $MoS_2$, unit parameters: a = 9.53 Å, b = 9.47 Å, γ = 120°. (b) CA SAM on $MoS_2$, unit parameters: a = 9.60 Å, b = 9.60 Å, γ = 120°. (b) CA·M SAM on $MoS_2$, unit parameters: a = 9.60 Å, b = 9.60 Å, γ = 120°.

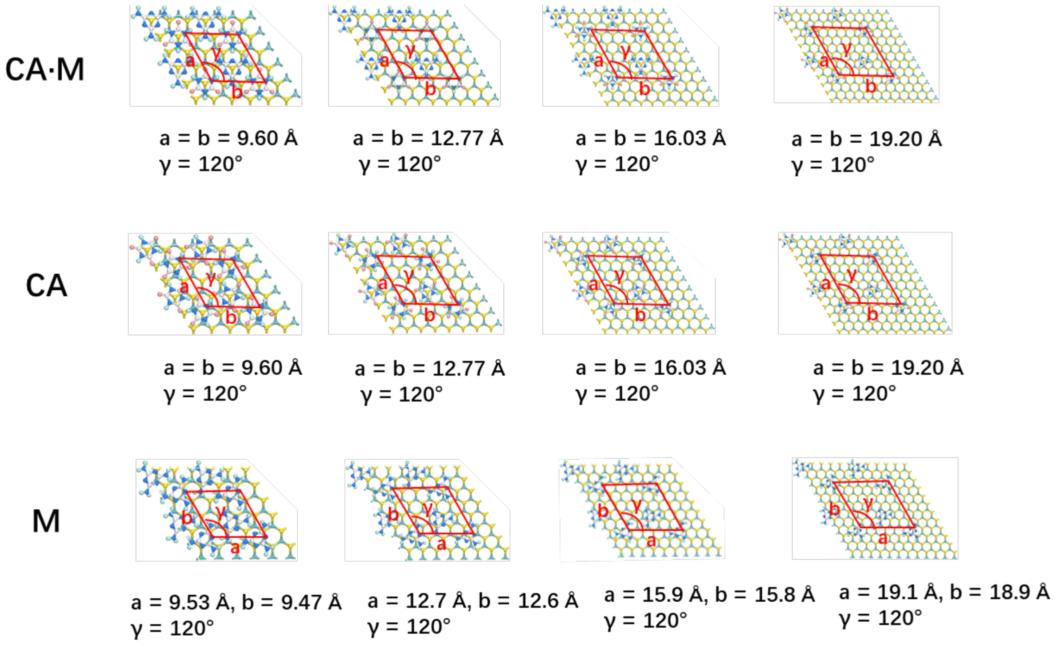


**Supplementary Figure 2: Lattice diffusion models for CA·M, CA and M self-assembly on monolayer $MoS_2$.**

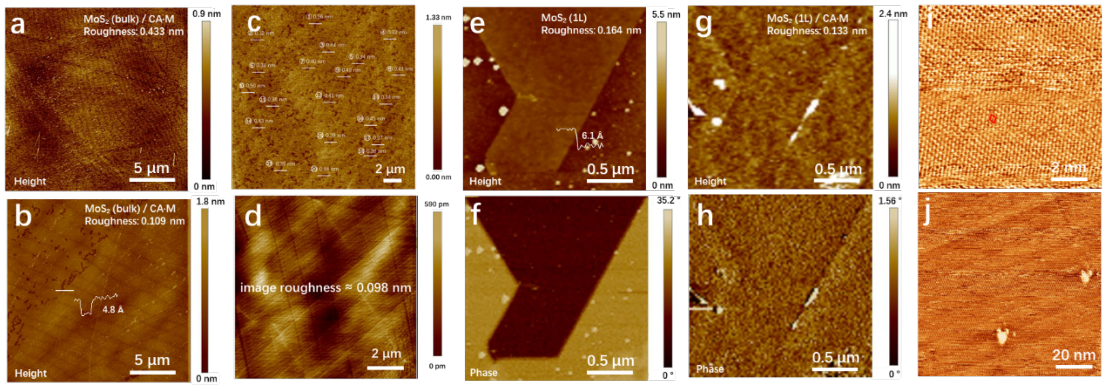


**Supplementary Figure 3: Morphological and structural characterization of CA·M 2D supramolecular network formation on $MoS_2$ surface.** AFM height images of CA·M SAM on $MoS_2$ bulk crystal surface: a) non-annealed and b) annealed at 430K in vacuum for 12h. c) Statistics of the thickness of CA·M molecular layer on sub-monolayer samples. d) the roughness of $MoS_2$ crystal surface. e) AFM height and corresponding f) phase image of mechanically exfoliated $MoS_2$ monolayer (1L) flake on Si / $SiO_2$ substrate. g) AFM height and corresponding h) phase image of CA·M-coated $MoS_2$ monolayer flake. (i) STM height image of 2H phase $MoS_2$ crystal surface. (j) Large area STM height image of CA·M network adsorbed onto $MoS_2$. Tunneling parameters: (i) $V_t$ = -430 mV, $I_t$ = 80 pA; (j) $V_t$ = -700 mV, $I_t$ = 55 pA.

Figure S3a displays the surface morphology a freshly cleaved $MoS_2$ bulk crystal coated with a bi-component CA·M film, processed via drop-casting followed by the solvent evaporation at room temperature. The AFM image shows that the molecular film is characterized by several sub-µm domains with boundaries appearing as dark lines for non-annealed samples. These fragmented small domains merge forming larger, micrometer-scaled structures upon thermal annealing at 430K for 12h in vacuum. As illustrated in Fig. S3b, a more uniform and less defective CA·M supramolecular network is obtained over a 400 $\mu m^2$ area, with only sporadic non-coated surface areas observed. The height profile performed along the coated $MoS_2$ surface (see white line on Fig. S3b) indicates that the CA·M film is monolayer, with thickness of ca. 4.8 Å. Moreover, the roughness of this CA·M supramolecular network decreased from 0.433 nm to 0.109 nm upon annealing. In order to quantify the thickness of the CA·M monolayer more accurately, we performed AFM topographical imaging of sub-monolayer thick films. Fig. S3c displays the statistics on the CA·M layer thickness executed by sampling data from 20 different sample' s regions; the resulting average value amounts to 0.397 ± 0.103 nm. Moreover, the bare $MoS_2$ surface has a roughness of about 0.1 nm, as depicted in Fig. S3d. Noteworthy, the distance between the two planar sheets in CA·M crystal has been previously estimated by AFM imaging as 0.317 nm. The difference in the CA·M monolayer thickness is mostly caused by the local environment. More precisely, while in our work the CA·M monolayer was physisorbed on $MoS_2$ surface hence it encountered strong molecule-substrate interactions, in the melamine–cyanuric acid crystal, the investigated CA·M layers are stacked via van der Waals forces. The use of different substrates can also lead to various adsorption distances, as our simulated vertical distance between CA·M molecular layer and $MoS_2$ surface is about 0.33 nm, while the simulated average distance between CA·M molecular layer and Au (111) surface has been reported being 0.371 nm.

The same CA M preparation was carried out on a mechanically exfoliated monolayer $MoS_2$ flake (1L) transferred onto the $SiO_2$ surface. The topographical images of pristine monolayer $MoS_2$ are displayed in Fig. S3e and S3f, which reveals a smooth surface (roughness 0.164 nm) having a thickness of 6.1 Å. The phase image indicates that the exfoliated $MoS_2$ surface is uniform and free of debris. After the CA·M SAM deposition, the $MoS_2$ flake surface is completely coated (roughness = 0.133 nm), as exhibited in Fig. S3g. The CA·M network also assembles outside the $MoS_2$ flake limits, i.e., on $SiO_2$, producing a uniform molecular film that covers the whole sample area, as shown in Fig. S3h. The STM image of 2H phase $MoS_2$ crystal surface is shown in Fig, S3i, and it displays a lattice constant of a = b = 3.14 Å and γ = 120°, in good accordance with theoretical lattice parameters. Fig. S3j is $10^4$ $nm^2$ STM image of $MoS_2$ / CA·M hybrid surface, which indicates M and CA molecules can form defect-free large-area domains on $MoS_2$ surface.

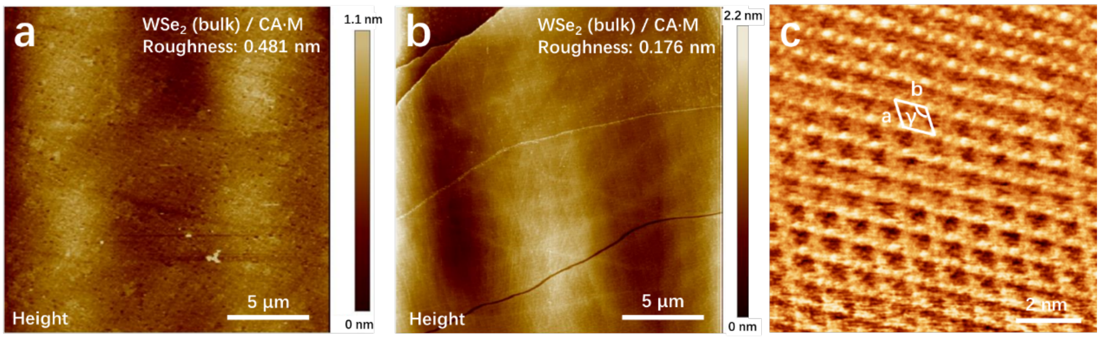


**Supplementary Figure 4: Morphological and structural characterization of CA·M 2D supramolecular network formation on $WSe_2$ surface.** AFM height image of CA·M molecular film deposited on $WSe_2$ crystal surface: a) non-annealed and b) after vacuum annealing at 430K for 12 h. (c) STM height image of CA·M co-assembled monolayer on $WSe_2$ surface after annealing. Tunneling parameters: $V_t$ = -820 mV, $I_t$ = 50 pA.

Likewise in films supported on $MoS_2$ surface, the formed CA M film is characterized by small molecular domains that connect with each other to form a continuous monolayer after vacuum annealing (Fig. S4a and S4b). STM measurements performed on such hybrid surfaces is illustrated in Figure S4c. Inset the unit cell is depicted, where the cell parameters are measured as a = b = 0.97 ± 0.05 nm, γ = 120 ± 2°. CA and M molecules pack on $WSe_2$ surface in the same manner as on $MoS_2$ surface (Fig. 2b&c in the main text).

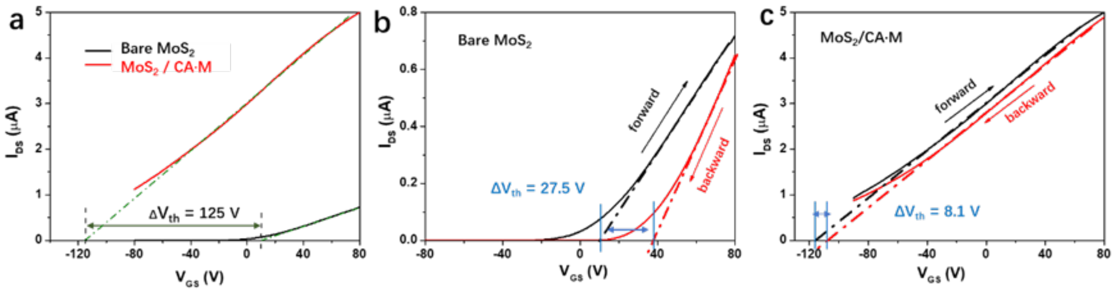


**Supplementary Figure 5: Transfer curve for monolayer $MoS_2$ field-effect transistor (FET),**

**at $V_{DS}$ = 100 mV, before and after CA·M surface assembly.** The device threshold voltage ($V_{TH}$) is calculated by extrapolating the current values to $I_{DS}$ = 0. (a) Transfer characteristics and corresponding $V_{TH}$ change caused by CA·M functionalization. (b-c) Transfer characteristics (reverse sweep) and respective hysteresis for FETs based on b) pristine $MoS_2$ c) and $MoS_2$ / CA·M vdW heterostructure.

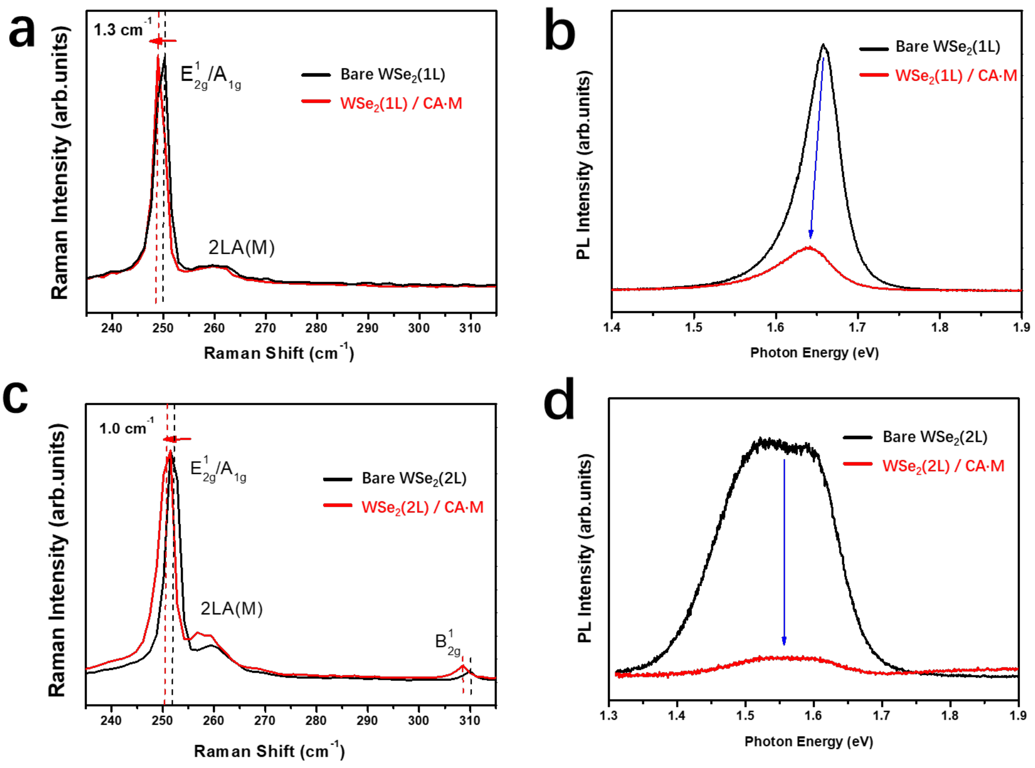


**Supplementary Figure 6: Raman and photoluminescence (PL) spectra of $WSe_2$ monolayer and bilayer flakes before (black curve) and after (red curve) CA·M coating.**

The $E^1_{2g}$ and $A_{1g}$ modes of $WSe_2$ are observed at approximately 250 $cm^{-1}$. These two modes are almost degenerate and cannot be easily resolved in the case of thin-layer $WSe_2$[3]. As seen in Fig. S6a, we only find a single $E^1_{2g}$ / $A_{1g}$ peak for the clean monolayer and bilayer flakes, where the peak position is slightly blue-shifted for bilayer $WSe_2$ in respect to monolayer $WSe_2$. Moreover, a broad side maximum at 260 $cm^{-1}$ (2LA(M)) is observed for the two samples. Finally, a small signature at 309 $cm^{-1}$ ($B^1_{2g}$) is evident in the Raman spectrum of bilayer $WSe_2$ (Fig. S6c). This can be assigned to the normally inactive $B_{2g}$ mode. Upon the CA·M SAM formation on the $WeS_2$ surface, the $E^1_{2g}$ / $A_{1g}$ band redshifts by 1.3 $cm^{-1}$ and 1.0 $cm^{-1}$ for monolayer and bilayer samples, respectively. Doping-induced Raman shift in $WSe_2$ flakes can be explained by the change of electron density. The n-type doping increases the electron-phonon scattering due to a higher electron concentration. Upon CA M coating, significant quenching is observed in the PL spectra of both monolayer and bilayer flakes, as seen in Fig. S6b and 6d. The PL peak is strongly suppressed and redshifted as a result of electron doping caused by the CA·M supramolecular lattice formed on the $WSe_2$ surface[4]. All these optical spectra were measured at room temperature in ambient.

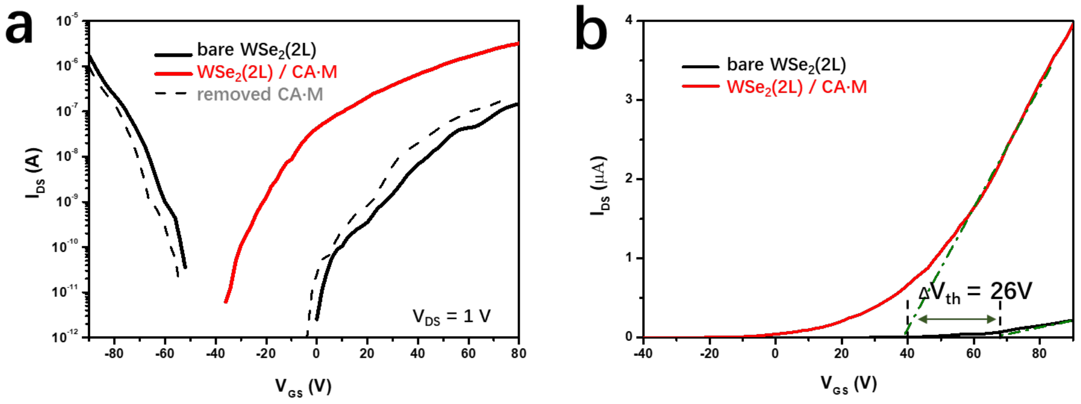


**Supplementary Figure 7: Transfer characteristic of bilayer $WSe_2$ FETs before and after CA·M coating, and removal.** (a) Reversibility of the $WeS_2$ FET transfer curves (semilog plot) upon CA·M assembly and removal. (b) $V_{th}$ shift in the transfer characteristics of bilayer $WeS_2$ FETs upon CA·M functionalization. $V_{DS}$ = 1V in both curves.

FETs based on bilayer $WSe_2$ have been produced to evaluate the CA·M doping. The as-prepared clean device (channel length / width is 2.31 μm / 3.49 μm) exhibits ambipolar transfer characteristics with slightly higher *p*-channel conductance, corresponding to close to mid-gap Schottky barriers at the source-drain Au contacts[5]. Upon deposition of the CA·M film, the bilayer $WSe_2$ device shows unipolar n-type transfer characteristics, with a $V_{TH}$ shift towards a more negative voltage (Fig. S7b). Applying a $V_{DS}$ of 1V, the field-effect mobility (μ) of bare $WSe_2$ is found amounting to 17.9 $cm^2V^{-1}s^{-1}$ for the holes and 0.5 $cm^2V^{-1}s^{-1}$ for the electrons. Whereas upon CA·M modification, μ amounts to 53.6 $cm^2V^{-1}s^{-1}$ for the electrons and tends to zero for holes. The significant improvement of electronic transport indicates that the CA·M SAM has a strong n-doping effect on $WSe_2$. From the observed $V_{th}$ shift of ca. 26 V, the n-type doping of $WSe_2$ corresponds to an electron density increase of 2.1 x $10^{12}$ $cm^{-2}$.

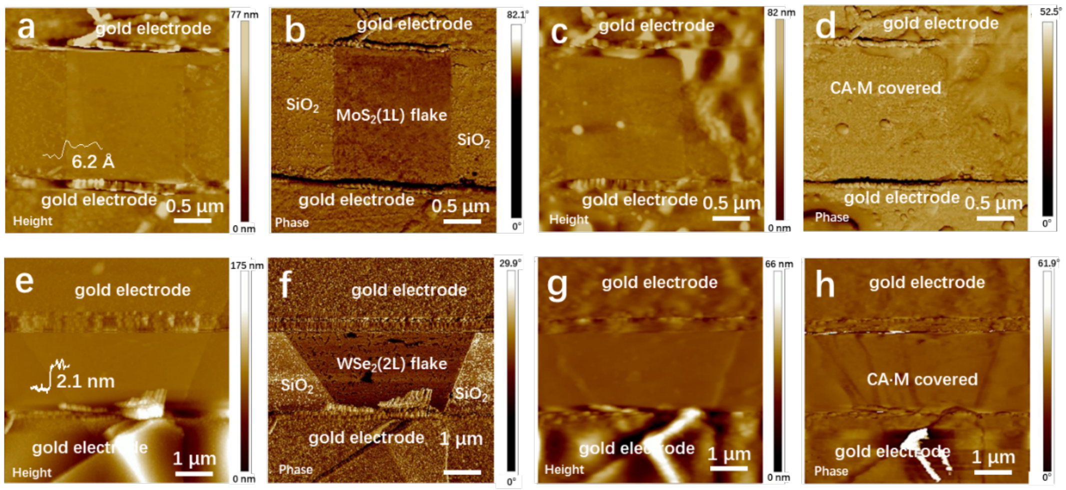


**Supplementary Figure 8: Morphological characterization of the FET channel based on monolayer $MoS_2$ and bilayer $WSe_2$.** (a) AFM height and (b) phase image of bare $MoS_2$

monolayer. (c) AFM height and (d) phase image of $MoS_2$ monolayer device covered with CA·M supramolecular lattice. (e) AFM height and (f) phase image of clean $WSe_2$ bilayer. (g) AFM height and (h) phase image of $WSe_2$ bilayer device coated with CA·M.

The AFM height profiles in Fig. S8a and S8e demonstrate that the flakes in our devices are respectively monolayer $MoS_2$ (thickness = 6.2 Å, roughness = 0.62 nm) and bilayer $WSe_2$ (thickness = 2.1 nm, roughness = 0.54 nm). The phase images exhibit a significant contrast between the $WSe_2$ flake and the $SiO_2$ surface, as seen in Fig S8b and S8f. Meanwhile, the smooth and uniform flake surface monitored in AFM images confirms that no contamination occurred during device fabrication. Upon the CA·M assembly on the surface, $MoS_2$ / CA·M and $WSe_2$ / CA·M heterostructures are formed. As shown in Fig S8c and S8g, the flakes in the device channel are completely coated with the CA·M SAM after vacuum annealing at 430K for 12h. No profound contrast difference exists between the coated areas, such as the TMDC flake, $SiO_2$ substrate, and Au electrode surfaces from the AFM phase images, revealing that the entire device area is uniformly functionalized with the CA·M SAM (Fig. S8d and S8h). The coated flakes in the devices exhibit a small surface roughness of ca. 0.81 nm for 1L-$MoS_2$ / CA·M and 0.68 nm for 2L-$WSe_2$ / CA·M.

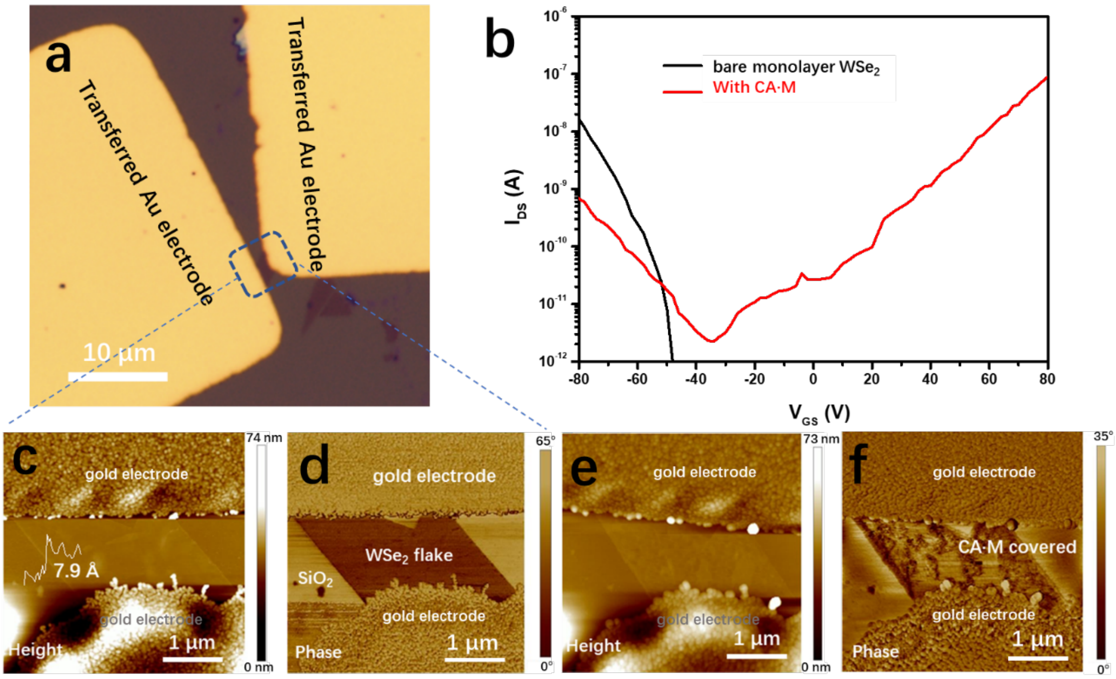


**Supplementary Figure 9: Monolayer $WSe_2$ FET device bearing Au nanomembrane source and drain electrodes produced by transfer method.** (a) Optical image of the as-prepared device. (b) transfer curves of the monolayer $WSe_2$ FETs before (black) and after (red) CA·M SAM attachment for $V_{DS}$ = 1V. AFM images of the device before (c,d) and after (e,f) CA·M supramolecular lattice attachment.

Devices prepared via photolithography typically require further high-temperature annealing to remove the residual solvent and photoresist, which can cause surface defects on $WSe_2$

surface[6]. Based on our experience, $WSe_2$ is much easier to be oxidized than $MoS_2$. Thus, to avoid this and obtain high-quality, stable p-type monolayer $WSe_2$ devices, dry transfer method of Au nanomembrane electrodes is a viable route[7]. Au electrodes (90 nm-thick) were pre-deposited on a Si / $SiO_2$ wafer and mechanically laminated on the TMDC crystal surface, resulting in an atomically flat metal-crystal interface[8]. Fig. S9a displays the topographic characteristics of the as-prepared device containing the transferred Au source and drain electrodes. The FET channel length (L) / width (W) is 1.6 µm / 2.6 µm for reported $WSe_2$ monolayer device. After depositing the CA·M mixture, the coated device is annealed at lower temperatures (373K rather than typical 430K) for 12h to remove adsorbed water and oxygen. The AFM phase image (Fig. S9e) indicates though that 373K is not high enough to form a continuous 2D crystalline CA M supramolecular film. On the boundaries of co-assembled molecular domains, some exposed areas are evident. We also observed that the surface roughness of $WSe_2$ monolayer exhibits a slight increase from 0.43 nm to 0.88 nm after partial coating with CA M. Back-gated monolayer $WSe_2$ FETs operated at $V_{DS}$ of 1V change their p-type behavior to ambipolar one (Fig. S9b), where the hole µ decreases from 8.2 $cm^2\ V^{-1}\ s^{-1}$ to 0.1 $cm^2\ V^{-1}\ s^{-1}$. The n-type transport after CA·M coating is characterized by electron µ of 7.14 $cm^2\ V^{-1}\ s^{-1}$. For monolayer $WSe_2$ FETs, one expects a more evident n-doping than for few-layer $WSe_2$ devices. Here, our results do not meet such expectations because of the poor crystallinity and coverage exhibited by CA·M SAM formed at lower annealing temperatures (373K) annealing that weakens the doping effect to some extent.

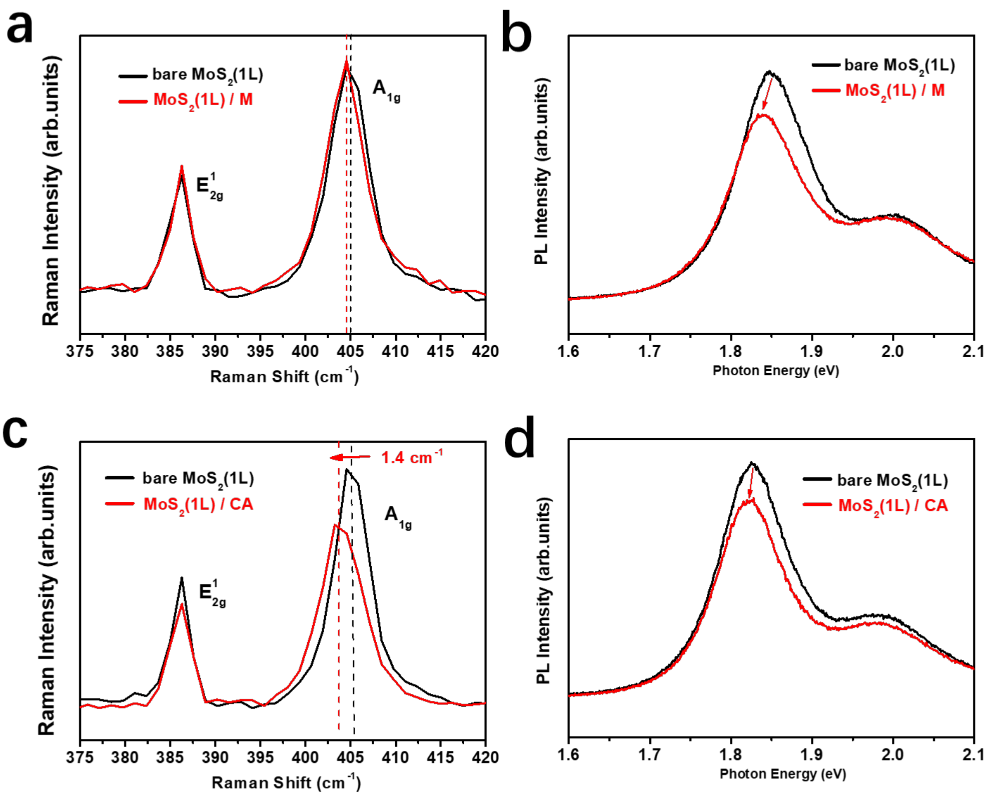


**Supplementary Figure 10: Raman (a,c) and PL (b,d) spectra acquired for monolayer $MoS_2$ flakes before (black curve) and after (red curve) coating with individual M and CA**

**molecules, measured at room temperature in ambient.**

Both individual CA and M molecules can dope (at a certain degree) the underlying monolayer $MoS_2$ flakes, as shown in Fig. S10. Raman spectroscopy measurements revealed a shortening of the distance between $E^1_{2g}$ and $A_{1g}$ peaks from 18.3 cm$^{-1}$ to 17.9 cm$^{-1}$ for M coating (Figure S10a), and to 16.9 cm$^{-1}$ for the CA coating (Figure S10c). Meanwhile, a marked quenching and redshift on the PL A exciton peak also confirm the n-doping of $MoS_2$ upon functionalization with the individual CA and M molecules (Fig. S10b and S10d).

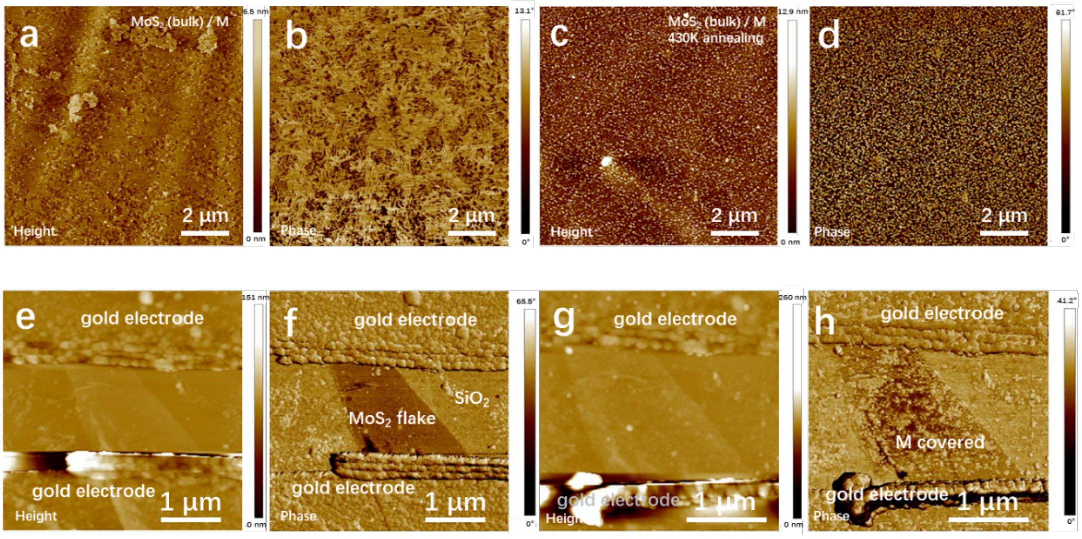


**Supplementary Figure 11: Morphological characterization of thin films made of M molecules on $MoS_2$ bulk and on monolayer $MoS_2$ FETs.** (a) AFM height and (b) phase images of M molecules deposited on $MoS_2$ bulk crystal surface before annealing. Respective (c) AFM height and (d) phase for the functionalized surface after vacuum annealing at 430K for 12h. (e) AFM height and (f) phase image of a $MoS_2$ monolayer before coating with M molecules. Respective (g) AFM height and (h) phase image after coating with M molecules and annealing.

As seen in Fig. S11a and S11b from AFM measurements, M molecules arrange themselves into small, sub-µm domains after casting and solvent evaporation, which is difficult to image by STM at ambient conditions. When annealed at 430K in vacuum, such a film structure is destroyed and the M molecules aggregate into smaller particles, as shown in Fig. S11c and S11d. The intramolecular hydrogen bonds between M molecules are disrupted at such elevated temperatures which does not occur for the network formed from the bi-assembled CA·M structure. The morphology of the monolayer $MoS_2$ device before and after M molecule deposition is shown in Fig. S11e-h. The surface roughness of the monolayer $MoS_2$ flake in the device channel increases significantly after coating, from 0.62 nm to 3.05 nm. Some exposed $MoS_2$ surface (darker regions in the AFM phase image) indicate that the on-surface coverage and the molecular film quality are significantly poor compared to the CA·M bi-assembled network.

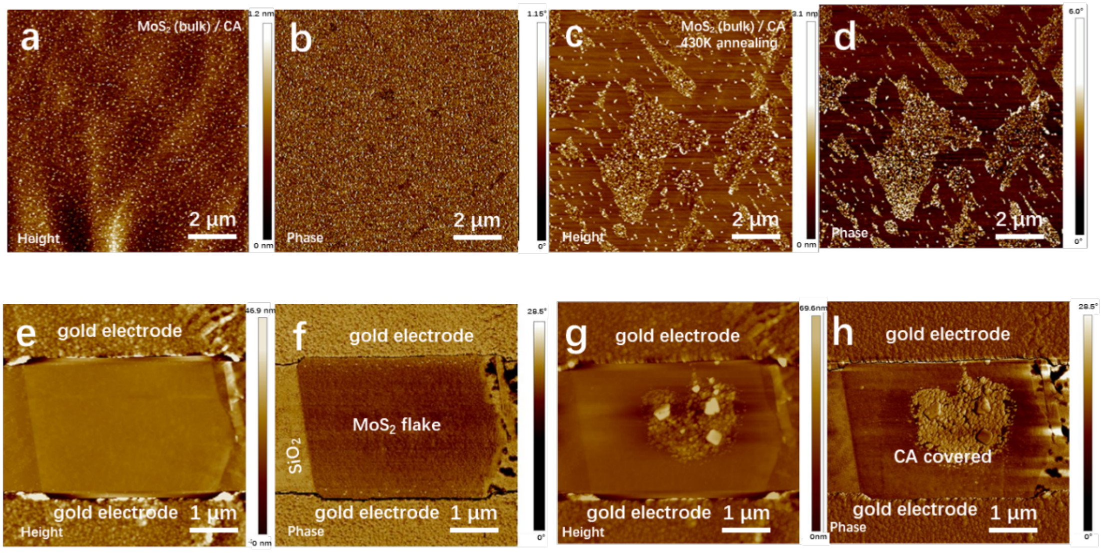


**Supplementary Figure 12: Morphological characterization of thin films made of CA molecules coated on $MoS_2$ bulk and on monolayer $MoS_2$ FETs.** (a) AFM height and (b) phase images of CA molecules deposited on $MoS_2$ bulk crystal surface before annealing. Respective (c) AFM height and (d) phase images for the functionalized surface after vacuum annealing at 430K for 12h. (e) AFM height and (f) phase image of a $MoS_2$ monolayer before coating with CA molecules. Respective (g) AFM height and (h) phase images after coating with CA molecules and annealing.

Fig. S12a and S12b show the AFM height and phase of bulk $MoS_2$ surface after coating with individual CA molecules, which is characterized by small, sub-µm aggregates. After vacuum annealing (430K, for 12h) the aggregates grow into islands, as seen in Fig. S12c and S12d. Onto the monolayer $MoS_2$ surface, CA molecules aggregate densely after vacuum annealing (Fig. S12g and S12h), leaving more than half of the device channel area exposed, i.e., non-coated. The surface roughness increases to 9.59 nm after coating with CA molecules, compared to 0.49 nm of the pristine $MoS_2$ surface.

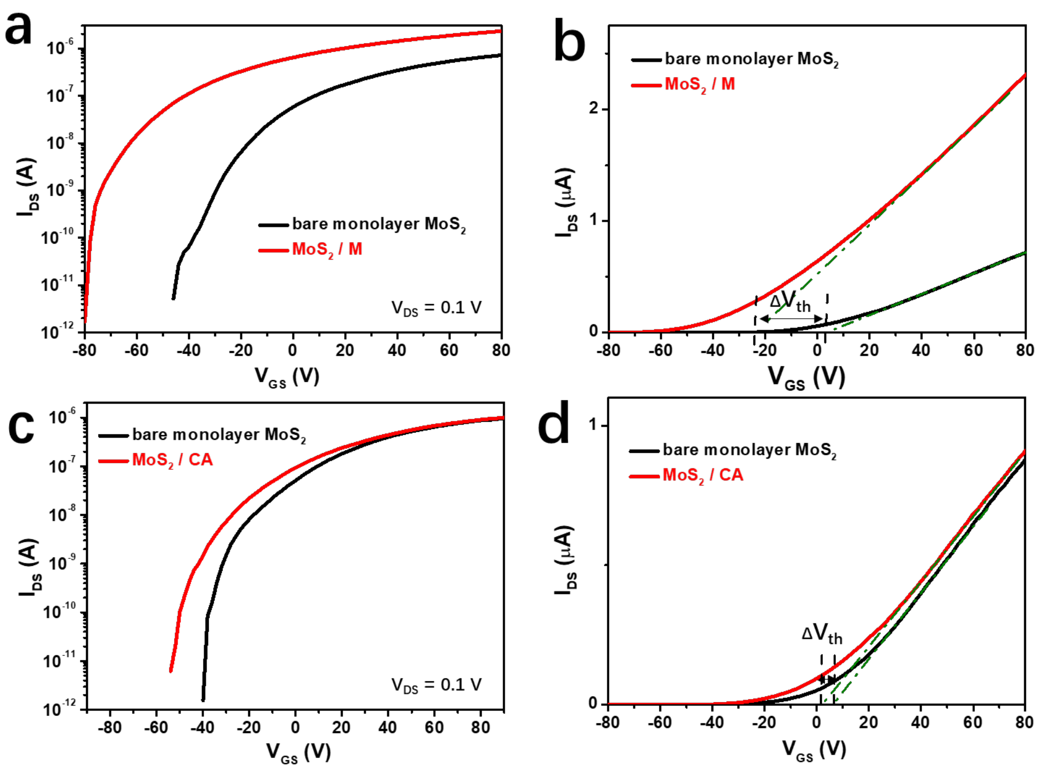


**Supplementary Figure 13: Transfer characteristic of monolayer $MoS_2$ FETs before and after film formation with individual CA and M molecules.** Monolayer $MoS_2$-based FET transfer curves before (black solid) and after (red solid) coating with M molecules as (a) semilog and (b) linear plots. Monolayer $MoS_2$-based FET transfer curves before (black solid) and after (red solid) coating with CA molecules as (c) semilog and (d) linear curves. The observed $\Delta V_{TH}$ shift upon coating with the individual molecules are indicate

The device employing M molecule deposition possesses a channel L / W of 1.46 μm / 1.22 μm, and the one used to evaluate the effects of CA deposition has a channel L / W of 2.96 μm / 4.01 μm. We observe a shift of the transfer curve towards more negative potentials, which is more marked when M molecules are used to cover the monolayer $MoS_2$ FET channel. The extracted electron μ increases from 9.3 $cm^2V^{-1}s^{-1}$ to 21.5 $cm^2V^{-1}s^{-1}$ upon deposition of M molecules (Fig. S13a), and from 7.5 $cm^2V^{-1}s^{-1}$ to 7.5 $cm^2V^{-1}s^{-1}$ after coating with individual CA molecules (Fig S13b). The negative shift of the FET transfer curves indicates that both M and CA molecules induce an n-doping effect to the underlying monolayer $MoS_2$. From the FET transfer characteristics (Fig. S13b and S13d), the doping intensity is calculated based on the $\Delta V_{TH}$. Here, after M coating the resulting $\Delta V_{th}$ is measured as 28.1 V, which corresponds to the electron density increases by $\Delta n = 2.2 \times 10^{12}$ $cm^{-2}$. Conversely, the $\Delta V_{TH}$ caused by CA molecule coating amounts to only 3.4 V, which leads to the small $\Delta n$ of $3.7 \times 10^{11}$ $cm^{-2}$. Notice that $\Delta n$ resulted from the CA·M co-assembly ($1.0 \times 10^{13}$ $cm^{-2}$, see main text) is approximately an order of magnitude larger than that for $MoS_2$ functionalization individual M molecules, and almost two orders of magnitude superior to that calculated for coatings with individual CA molecules.

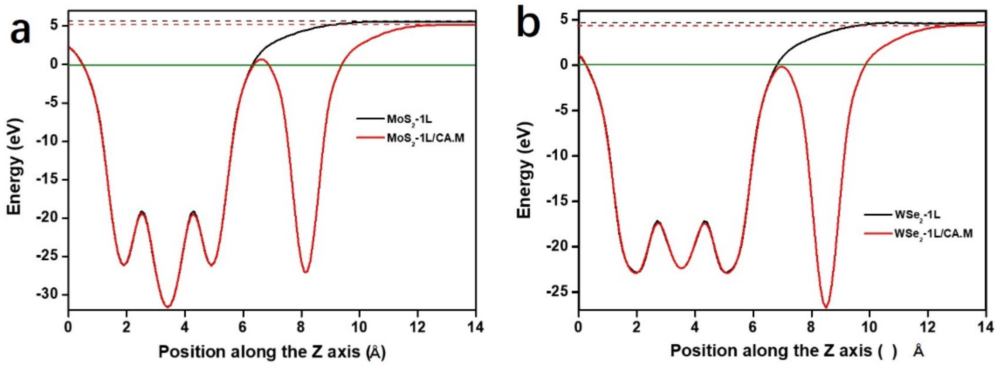


**Supplementary Figure 14: Work function simulation.** WF plots of (a) pristine $MoS_2$ surface and CA·M coated $MoS_2$ surface, (b) pristine $WSe_2$ and CA·M coated monolayer $WSe_2$ surface.

As shown in the WF plots, two asymptotic regions can be distinguished: the left asymptotic region, which allows one to calculate the WF for pristine $MoS_2$ or $WSe_2$ surface; and the right asymptotic region, which enables estimation of the WFs for the SAM covered surfaces. The calculated WF values are 5.23 eV for $MoS_2$ / CA·M, 5.45 eV for $MoS_2$, and 6.07eV for CA·M SAM. The negatively shifting in WF is 0.22 eV. Corresponding WF results are obtained in $WSe_2$ / CA·M hybrid structure: 4.38 eV for $WSe_2$/CA·M and 4.55 eV for pristine $WSe_2$ surface. Similar as on $MoS_2$, the negative shifting of 0.17 eV indicates an *n*-type doping effect of CA·M network.

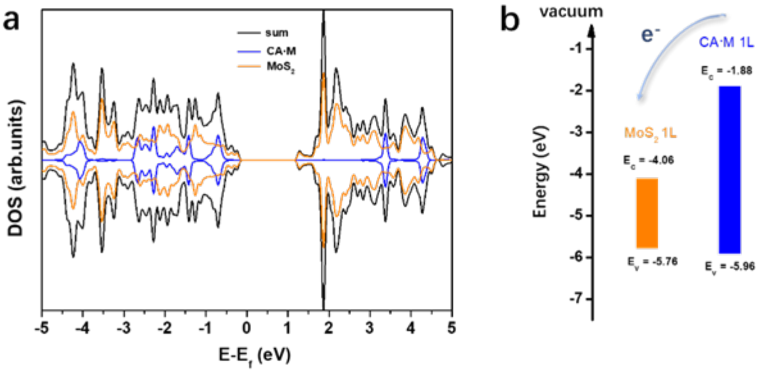


**Supplementary Figure 15: Energy levels simulation.** (a) Partial density of states (PDOS) plots of monolayer $MoS_2$ coated with the CA M supramolecular network. (b) Energy band alignment of $MoS_2$ and CA·M monolayer.

The charge transfer from CA·M to $MoS_2$ has a direct impact on the energy levels of the hybrid assembly. The band gap ($E_g$) of the CA·M co-assembled network is approximately 4.08 eV, while for the $MoS_2$ / CA·M hybrid structure $E_g$ is calculated as 1.48 eV. The interaction between the CA·M network and the $MoS_2$ surface leads to an overlap between states, and further reduction of the $MoS_2$ monolayer band gap by approx. 0.22 eV.

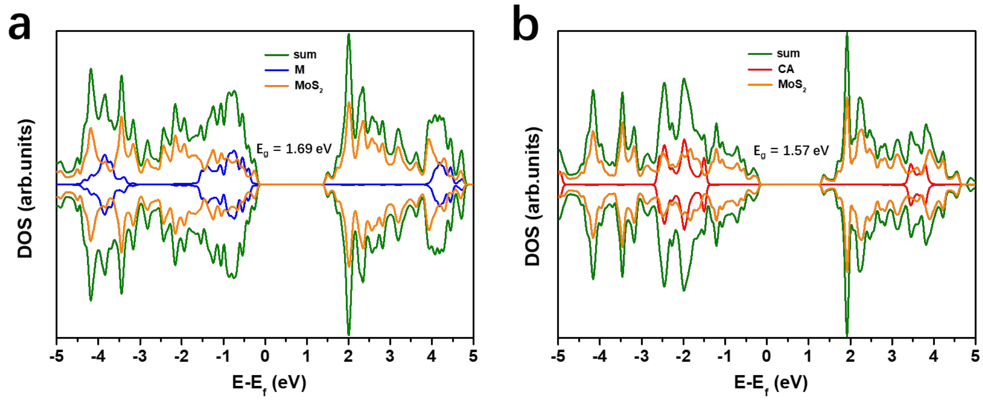


**Supplementary Figure 16: Partial density of states (PDOS) plots of monolayer $MoS_2$ coated with (a) CA and (b) M supramolecular network.**

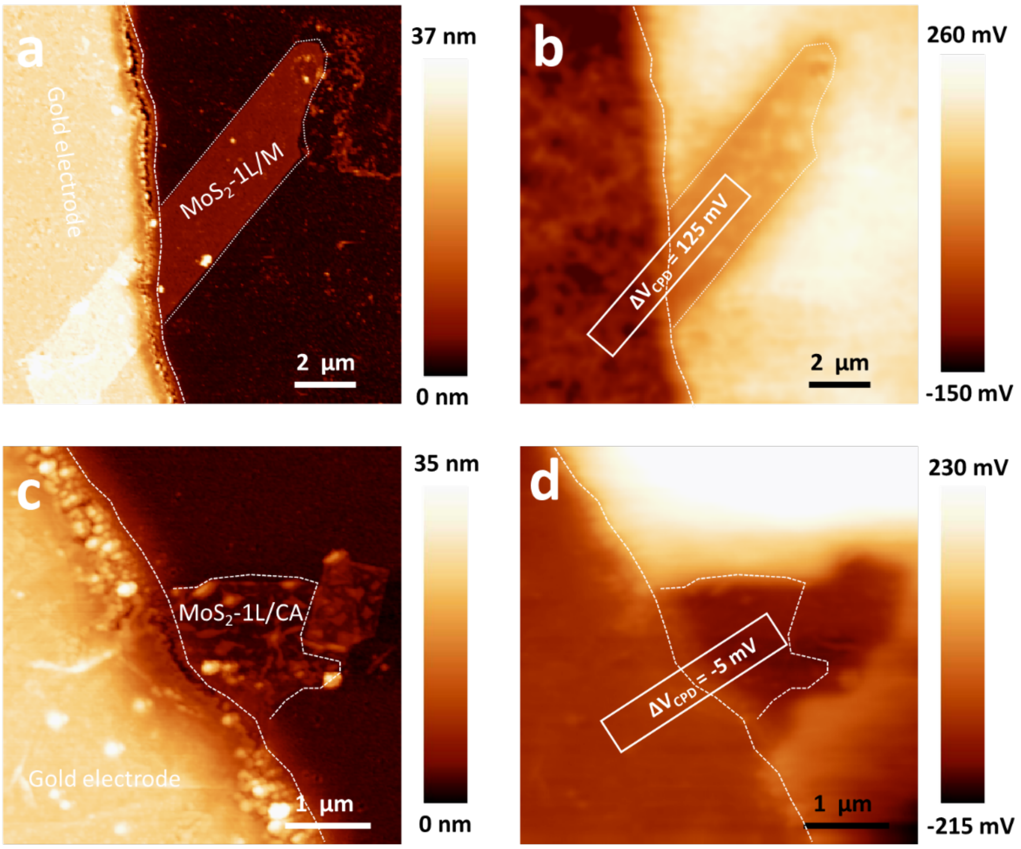


**Supplementary Figure 17: KPFM measurements on thin films made of individual M and CA molecules on monolayer $MoS_2$ flakes.** (a) AFM height and (b) KPFM images of M-coated monolayer $MoS_2$ flake. (c) AFM height and (d) KPFM images of CA-coated monolayer $MoS_2$ flake. The experimental surface potential (SP) of the $MoS_2$ flake and the Au electrode is obtained by averaging values from the marked regions.

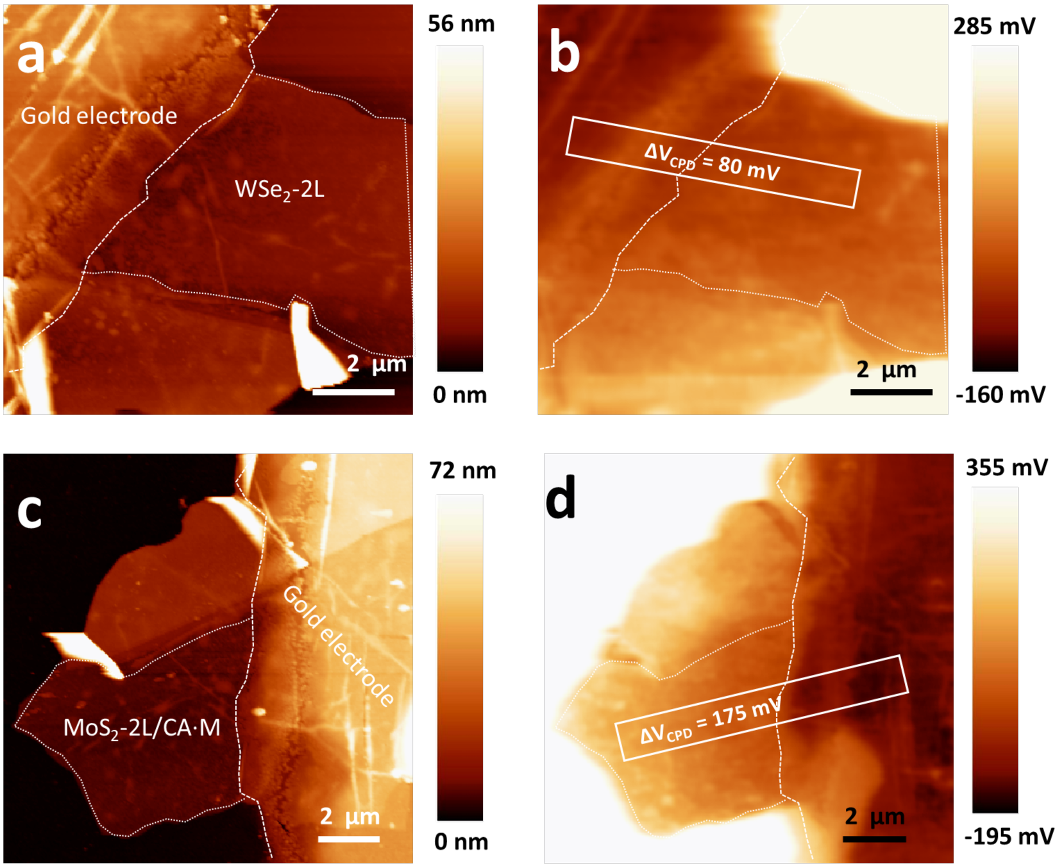


**Supplementary Figure 18: KPFM measurements on bilayer $WSe_2$ flake before and after CA·M modification.** (a) AFM height and (b) KPFM images of bilayer $WSe_2$ flake. (c) AFM height and (d) KPFM images of CA·M-functionalized $WSe_2$ bilayer flake. The experimental SP value of the $WSe_2$ flake and the Au electrode is obtained by averaging the values at the marked regions.

**Supplementary Table 1: Experimental WF based on KPFM.**

| Material | WF (eV) | N° of samples |
|---|---|---|
| $MoS_2$-1L | 4.80 ± 0.02 | 5 |
| $MoS_2$-1L/CA·M | 4.68 ± 0.03 | 6 |
| $MoS_2$-1L/M | 4.72 ± 0.02 | 5 |
| $MoS_2$-1L/CA | 4.83 ± 0.05 | 5 |
| $WSe_2$-2L | 4.77 ± 0.02 | 5 |
| $WSe_2$-2L/CA·M | 4.66 ± 0.05 | 8 |

The WF can be estimated from the measured SP difference between TMDC flake and Au electrode. As discussed in the main text, here we assume that the WF of polycrystalline Au at ambient conditions is $WF_{Au}$ = 4.85 eV, as determined via Kelvin probe force microscopy on macroscopic Au thin films produced via thermal evaporation. The WF of our flakes are calculated by using the equation: $WF_{FLAKE} = WF_{Au} + e\Delta V_{CPD}$[9].

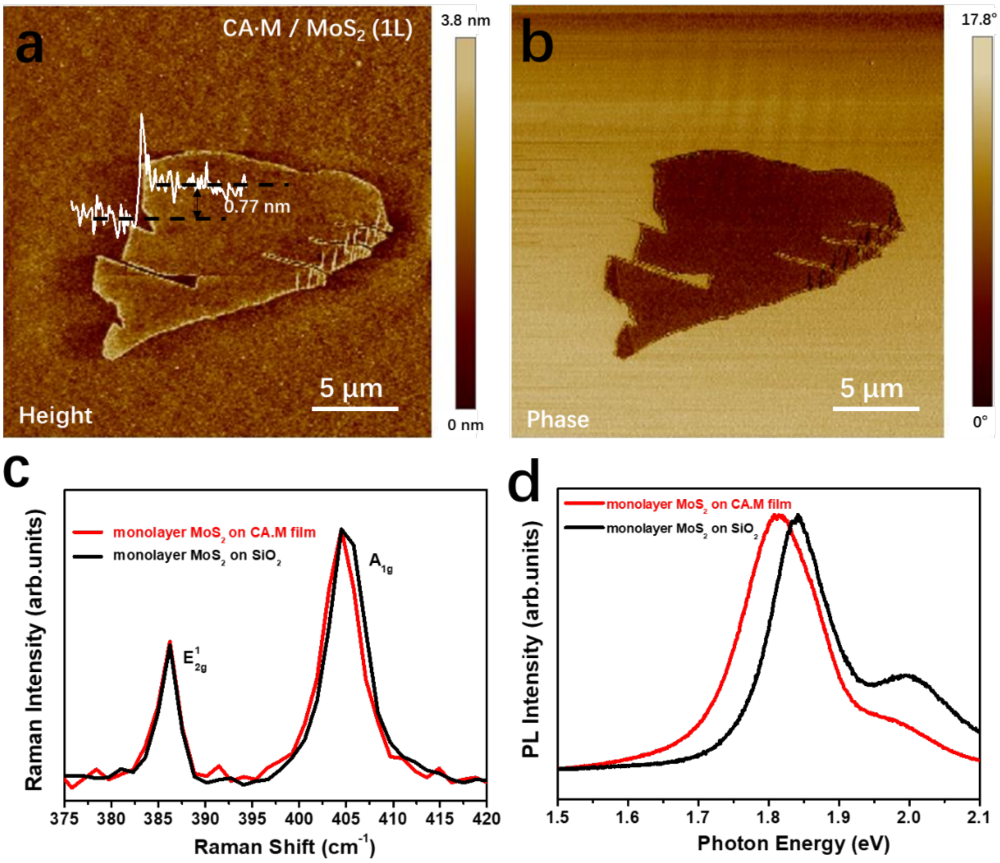


**Supplementary Figure 19: monolayer $MoS_2$ flake deposited on CA·M supramolecular network.** (a) AFM height and (b) phase image. (c) Normalized Raman spectra obtained for CA·M / $MoS_2$ hybrid surface compared with monolayer $MoS_2$ flake on $SiO_2$. (d) Corresponding normalized PL spectra

Coating the whole $SiO_2$ surface (1 $cm^2$) with a continuous CA M monolayer produced via drop-casting is not feasible. In this sense, multilayer CA·M films are preferred to serve as a buffer layer between $SiO_2$ substrates and the transferred TMDC flakes. Here, multilayer CA M is obtained by drop-casting 100 µL of 1 X $10^{-6}$ M CA and M mixture on a preheated $SiO_2$ wafer (430 K), and further annealed at 450K for 12h. Transferred monolayer $MoS_2$ flake adheres well on CA·M film by mechanical exfoliation, forming a CA·M / $MoS_2$ hybrid structure. As seen in AFM height image (Fig. S19a), the $SiO_2$ / CA·M surface is smooth with roughness ca. 0.29 nm, while the attached monolayer $MoS_2$ flake exhibits roughness of 0.28 nm. The flake thickness is measured as 0.77 nm, confirming its monolayer nature. AFM phase image reveals an excellent contrast between CA M and $MoS_2$ (Fig. S19b). Fig. S19c shows the normalized the Raman spectra for monolayer $MoS_2$ onto CA·M film and onto $SiO_2$. The $MoS_2$ $A_{1g}$ peak shows a slightly redshift. The underlayer CA·M film is able to n-dope the upper layer monolayer $MoS_2$, likewise the doping effect observed when CA M monolayer is deposited on top of the $MoS_2$ flake. The n-doping effect is also confirmed by the normalized PL spectroscopy, as shown in Fig. S19d. Compared with data from $MoS_2$ transferred to bare $SiO_2$, the PL spectra of monolayer $MoS_2$ on CA·M film reveals a marked redshift at A excitation peak and a wider peak width.

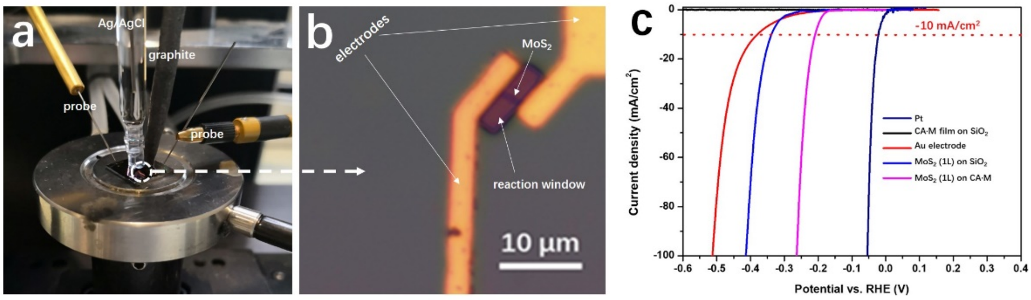


**Supplementary Figure 20: Hydrogen evolution reaction (HER) measurements on monolayer $MoS_2$ flakes.** (a) Photograph of the micro-electrochemical cell. (b) Optical image of the as-prepared device showing the photoresist window for electrolyte confinement. (c) The HER polarization curves of blank sample, Au electrode, monolayer $MoS_2$, monolayer $MoS_2$ on CA·M thin film, and Pt.

Fig. S20a illustrate the device schematics and experimental arrangement used to perform HER measurements. Fig. S20b shows that the Au working electrode (WE) well connects the $MoS_2$ flake while being passivated by a photoresist layer to avoid the leakage currents across the chip. A window in the photoresist is open by optical lithography, so the electrolyte can access only the device' s active region (connected $MoS_2$ flake). Blank control group has been fabricated without having a $MoS_2$ flake, i.e. only the CA·M film as WE. As shown in Fig. S20c, no catalytic signal is observed for the CA·M film, and the overpotentials (η) at a catalytic current density of 10 mA/cm$^{-2}$ for Au, Pt, $MoS_2$, and CA·M / $MoS_2$ surface are 382, 20, 340, and 208 mV, respectively.

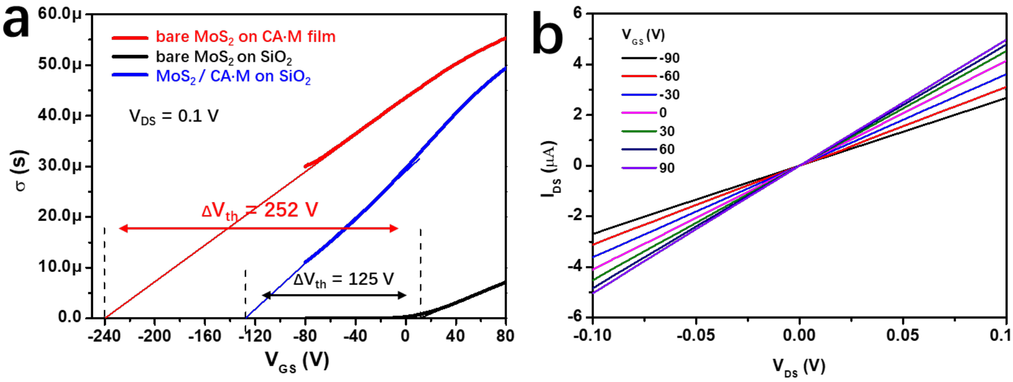


**Supplementary Figure 21: Electrical characteristics of monolayer $MoS_2$ FETs on CA·M film.** (a) FET transfer curves. (b) Output FET curves for monolayer $MoS_2$ on CA·M multilayer film, L = 1.95 μm and W = 1.69 μm.

As the buffer CA·M thin film can also n-dope the upper $MoS_2$ flake, back-gated FETs were fabricated to probe their electrical characteristics. Fig, S21a shows the transfer characteristics of monolayer $MoS_2$ FETs. The device transfer curves expressed in terms of the channel

conductivity (σ) facilitates the comparison between the two devices fabricated independently. The negative $V_{TH}$ shift in respect to the FET response of monolayer $MoS_2$ on $SiO_2$ (red curve) indicates that the CA·M / $MoS_2$ hybrid device exhibits even larger doping (larger $\Delta V_{TH}$ shift) than $MoS_2$ / CA·M devices. The possible explanation is that multilayer CA·M film (used in CA·M / $MoS_2$ devices) is more effective to dope the TDMC than the monolayer CA·M. The electron density change (Δn) induced by the CA·M network is calculated as 1.1 x $10^{13}$ $cm^{-2}$ for monolayers deposited on top of $MoS_2$ and 2.0 x $10^{13}$ $cm^{-2}$ for thin films underneath the 2DM. The output curves in Fig. S21b indicate the formation of good electrical connection (Ohmic contact) for between Au and $MoS_2$ for both CA·M / $MoS_2$ hybrid FET devices even under a small $V_{DS}$.

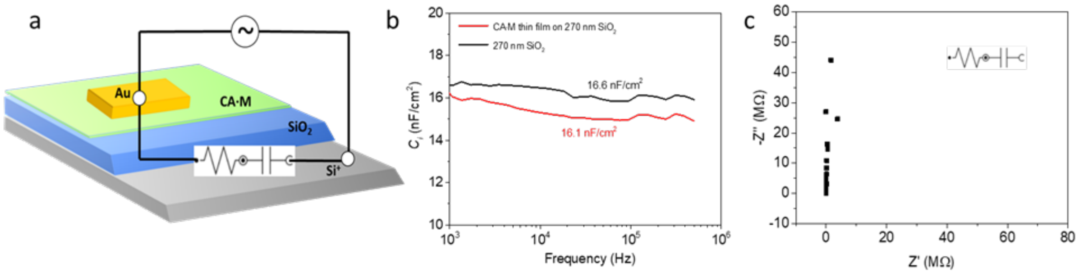


**Supplementary Figure S22. The capacitance of the dielectric layer with / without CA·M thin film** (a) schematic of the capacitance measurement. (b) Specific capacitance ($C_i$) of the device $SiO_2$ insulating layer with / without the CA·M thin film on 270 nm $SiO_2$. (c) Nyquist plot of electrochemical impedance spectroscopy (EIS) for the device with CA·M thin film on 270 nm $SiO_2$, and inserted is the equivalent circuit.

The capacitance of the dielectric layer with / without CA·M thin film is measured by Metrohm Autolab PGSTAT128N potentiostat. in the frequency (f) range of 1000 Hz to 500 kHz. The applied signal is a sine wave, with 0.01 V root mean square voltage (Vrms) amplitude and 0 V DC bias. The $SiO_2$ has a thickness of 270 nm and corresponding capacitance of 16.6 $nF/cm^2$. The capacitance is calculated with the following equation.

$$C = Z'/2\pi f|Z|^2$$

The capacitance remains constant within the analyzed frequency range. After the deposition of 5-10 nm CA·M molecular film, the capacitance of the dielectric layer is slightly decreased to 16.1 $nF/cm^2$, which is a neglectable change. As the thickness of the CA·M thin film is about 5 nm (10 layers), the capacitance of the 270 nm $SiO_2$ substrate has almost no change with and without the CA·M film attachment.

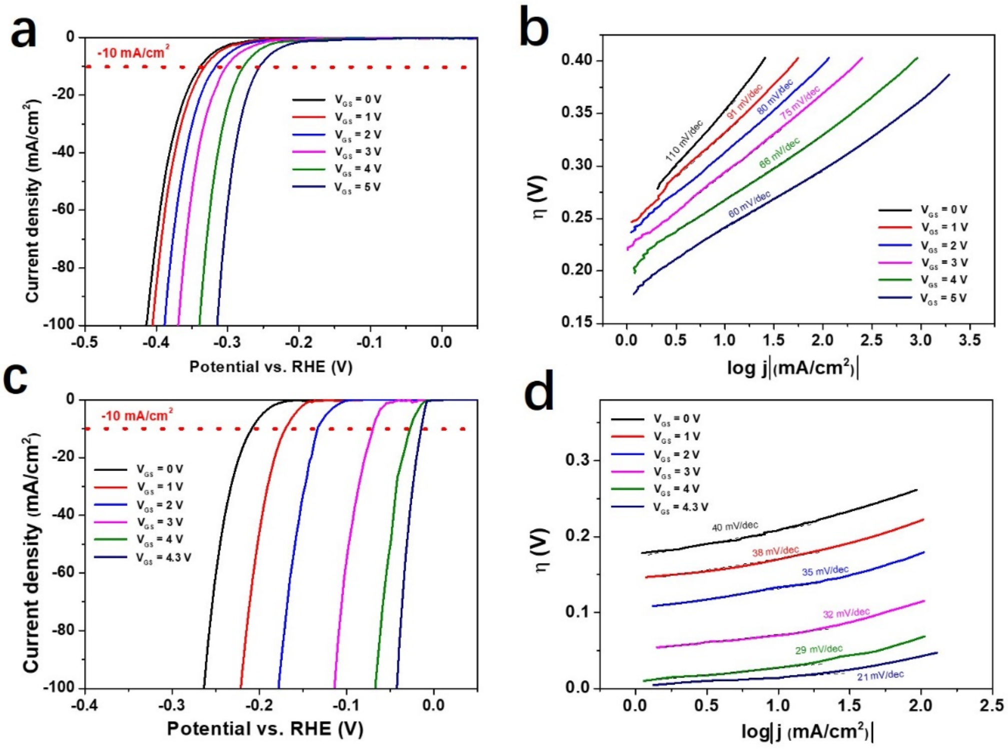


**Supplementary Figure 23: Polarization curves for HER measurements and corresponding Tafel plots.** $MoS_2$ monolayer flake on (a,b) $SiO_2$ and (c,d) on CA·M film at different back gate voltages ($V_{GS}$).

The HER polarization curves for monolayer $MoS_2$ onto $SiO_2$ devices under $V_{GS}$ varying from 0 to 5 V are shown in Fig. S23a. The curves shift towards smaller potentials while increasing $V_{GS}$, indicating that the sample catalytic property is largely improved upon gate bias. A relatively low overpotential (η) of 255 mV for $V_{GS}$ = 5V is achieved with respect to the scenario where $V_{GS}$ is null (η = 347 mV). Fig. S23b shows the corresponding Tafel slopes that decrease from 110 mV dec$^{-1}$ to 60 mV dec$^{-1}$ upon $V_{GS}$ application. Fig. S23c and S23d display the field-tuned polarization curves and the related Tafel slopes for CA·M / $MoS_2$ hybrid surface. The catalytic property of the hybrid device is largely boosted when $V_{GS}$ increase from 0 V to 4.3 V. At $V_{GS}$ = 4.3V, η reaches as low as 14 mV, which is comparable with that of Pt. Because of the rapid discharge process on the CA·M / $MoS_2$ hybrid surface, it achieves smaller Tafel slopes (29 mV dec$^{-1}$) than Pt for $V_{GS}$ > 4V. All η and Tafel slopes are summarized in Table S2 and S3.

**Supplementary Table 2: HER overpotential at different gate voltages ($V_{GS}$).**

| $V_{GS}$ (V) / η (mV) | 0 | 1 | 2 | 3 | 4 | - |
|---|---|---|---|---|---|---|
| Monolayer $MoS_2$ on $SiO_2$ | 340 | 332 | 318 | 302 | 278 | 255 (5V) |
| Monolayer MoS2 on CA·M molecular film | 208 | 171 | 134 | 70 | 28 | 14 (4.3 V) |

**Supplementary Table 3:** Tafel slope at different gate voltages ($V_{GS}$).

| $V_{GS}$(V) / Tafel slope (mV dec$^{-1}$) | 0 | 1 | 2 | 3 | 4 | - |
|---|---|---|---|---|---|---|
| Monolayer $MoS_2$ on $SiO_2$ | 110 | 91 | 80 | 75 | 66 | 60 (5 V) |
| Monolayer $MoS_2$ on CA·M molecular film | 40 | 38 | 35 | 32 | 29 | 21 (4.3 V) |

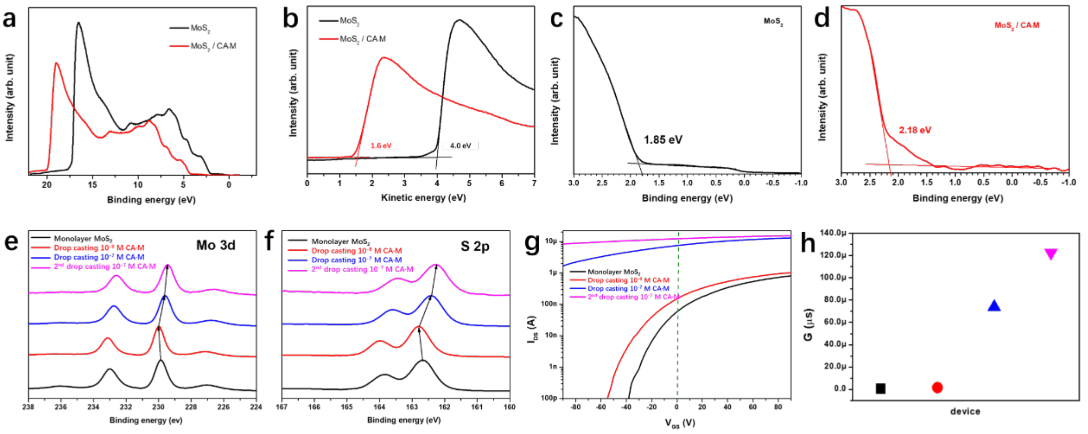


**Supplementary Figure 24: Photoelectron spectroscopies analysis:** (a) UPS spectra of monolayer CVD $MoS_2$ before and after CA·M film attachment. (b) low kinetic energy (secondary electron cutoff) region. (c) low binding energy region (near the $E_F$) of (c) $MoS_2$ and (f) $MoS_2$ / CA·M. XPS core-level spectra of (e) Mo 3d and (f) S 2p as a function of CA·M thickness on monolayer MoS2. (g) Transfer characteristics of monolayer MoS2 device with increasing CA·M film from sub-monolayer to around bilayer, $V_{DS}$ = 0.1 V. (h) Electric conductance at Vg = 0 V based on the device shown in (c).

To have a better understanding of the n-doping mechanism of $MoS_2$ by CA·M layer attachment, ultraviolet photoelectron spectroscopy (UPS) and X-ray photoelectron spectroscopy (XPS) measurements are performed to study the interfacial electronic structure at the monolayer CVD $MoS_2$ / CA·M heterostructure interface. The comparison of UPS spectra before and after CA·M film (about 2 nm thick) deposition is shown in Fig. S24a. By linearly extrapolating the low kinetic energy onset, the work function of monolayer $MoS_2$ sharply decreases from 4.0 eV to 1.6 eV (Fig. S24b), originating from substantial interfacial electron transfer from CA·M film to $MoS_2$. At the low energy region, the work function reduction is accompanied by a downward band-bending of the valence band of $MoS_2$ toward higher binding energy. In the low binding energy region of UPS spectra (Fig. S24c and Fig. S24d), the binding energy at valence band maximum upshift by 0.33 eV (from 1.85 eV to 2.18 eV) away from Fermi level ($E_F$). Fig. S24e and Fig. S24f show the Mo 3d and S 2p core levels evolved with the increased CA·M coverage. At the initial stage, with sub-monolayer coverage

(drop-casting 1 x $10^{-8}$ M solution), the binding energies of Mo 3d and S 2p core levels slightly increased by around 0.15 and 0.13 eV, respectively. This indicates that the Fermi level of $MoS_2$ shifts toward the conduction band, hence confirming the effective electron doping process. With an increase in the CA·M coverage to a complete monolayer (drop-casting 1 x $10^{-7}$ M solution), the binding energies of Mo 3d and S 2p core levels significantly decrease by about 0.26 and 0.27 eV, respectively. And a further increase in the CA·M thickness to around bilayer ($2^{nd}$ drop-casting 1 x $10^{-7}$ M solution), the decrease of binding energies can reach 0.40 for Mo 3d and 0.41 eV for S 2p. The decrease of the core level energy is caused by the extra electron-induced phase transition of TMDs[10]. The transfer curve (Fig. S24g) demonstrates that the CA·M coated monolayer $MoS_2$ exhibits n-type doping characteristics. Moreover, when the channel is fully covered with CA·M film (drop-casting 1 x $10^{-7}$ M solution), very weak gate dependence is observed, revealing a transition from the semiconducting phase to the metallic phase. Herein, we have calculated the electron conductance ($G = I_{SD}/V_{SD}$) at $V_{GS}$ = 0 V with the increased CA·M thickness, as shown in Fig. S24h. The electron conductance of monolayer $MoS_2$ increase from 0.6 to 130 µS, which confirms the strong electron doping on $MoS_2$.

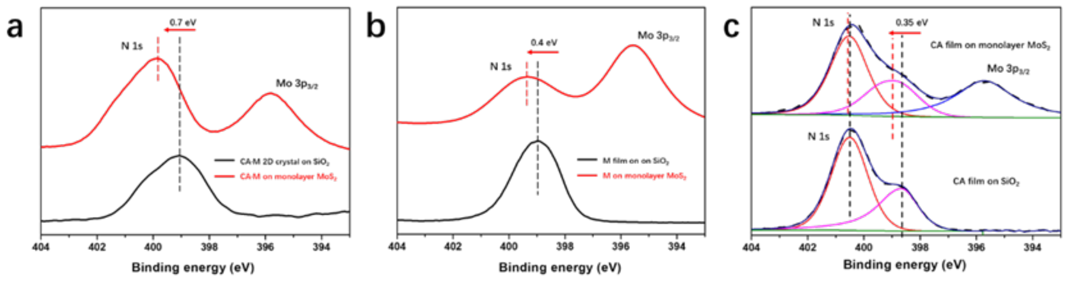


**Supplementary Figure 25: N 1s XPS core level spectra** for (a) $MoS_2$ / CA·M, (b) $MoS_2$ / M and (c) MoS2 / CA interface.

To assess the behaviors of molecular film in the charge transfer processes, core level spectra (XPS) of monolayer CVD $MoS_2$ with sub-monolayer molecular coverage are analyzed (Fig. S25). Note, the N 1s level is related to CA and M molecules. Compared with molecular film deposition on $SiO_2$, the N 1s core-level shift to higher energy for all three interfaces, which dominantly represents the process of losing electrons from CA and M molecules owing to the interfacial charge transfer[11]. The $MoS_2$ / CA·M interface has the most obvious shift of N 1s by 0.7 eV, compared with 0.4 eV for $MoS_2$ / M interface and 0.35 eV for $MoS_2$ / CA interface. In case of CA, the N 1s XPS spectra could be deconvoluted into two peaks, as seen in Fig. S25c. The peak around 400.5 eV is assigned to the nitrogen of CA tautomer in the form of triketone, whereas the peak around 398.7 eV relates to the nitrogen of CA tautomer in form of triacid. The charge transfer is more obvious when the CA molecule is adsorbed on $MoS_2$ surface in triacid form. Therefore, for all three systems, the N 1s core-level spectra consistently confirm the occurrence of electron transfer from molecule layer to $MoS_2$.

## Supplementary References


1. Musumeci, D & Ward, M. D. Elucidation of the crystal growth mechanism of melamine-cyanuric acid by using real time in situ atomic force microscopy. *CrystEngComm*, **13**, 1067–1069 (2011).
2. Zhang, H. et al. One-Step Preparation of Large-Scale Self-Assembled Monolayers of Cyanuric Acid and Melamine Supramolecular Species on Au(111) Surfaces. *J. Phys. Chem. C* **112,** 4209-4218 (2008).
3. Zhao, W. et al. Lattice dynamics in mono- and few-layer sheets of WS2 and WSe2. *Nanoscale* **5**, 9677-9683 (2013).
4. Ji, H.G. et al. Chemical Doping: Chemically Tuned p- and n-Type WSe2 Monolayers with High Carrier Mobility for Advanced Electronics. *Adv. Mater.* **31**, 1970301 (2019).
5. Schulman, D.S., Arnold, A.J. & Das, S. Contact engineering for 2D materials and devices. *Chem. Soc. Rev.* **47**, 3037-3058 (2018).
6. Stoeckel, M.-A. et al. Boosting and Balancing Electron and Hole Mobility in Single- and Bilayer WSe2 Devices via Tailored Molecular Functionalization. *ACS Nano* **13**, 11613-11622 (2019).
7. Liu, Y. et al. Approaching the Schottky–Mott limit in van der Waals metal–semiconductor junctions. *Nature* **557**, 696-700 (2018).
8. Tang, Q. et al. Micrometer- and Nanometer-Sized Organic Single-Crystalline Transistors. *Adv. Mater.* **20**, 2947-2951 (2008).
9. Hansen, W.N. & Hansen, G.J. Standard reference surfaces for work function measurements in air. *Surf Sci* **481**, 172-184 (2001).
10. Lie, B. et al. Direct Observation of Semicobductor – Metal Phase Transition in Bilayer Tunsten Diselende induced by Potassium Surface Functionalization. *ACS Nano* **12,** 2070-2077 (2018).
11. Wang, X. et al. Interfacial Covalent Bonds Regulated Electron-Deficient 2D Black Phosphorous for Electrocatalytic Oxygen Reactions. *Adv. Mater.* **33,** 2008752 (2021).
